# NurseVerse: nursing in the metaverse era

**DOI:** 10.3389/fmed.2025.1577153

**Published:** 2025-07-04

**Authors:** Xia Li, Yaoqun Zhou, Miao Xue

**Affiliations:** ^1^General Practice Ward/International Medical Center Ward, General Practice Medical Center, West China Hospital, Sichuan University, Chengdu, Sichuan, China; ^2^West China School of Medicine, West China Hospital, Sichuan University, Chengdu, China; ^3^West China Hospital, Sichuan University, Chengdu, Sichuan, China; ^4^General Practice Medical Center, West China Hospital, Sichuan University, Chengdu, Sichuan, China

**Keywords:** metaverse, virtual reality, augmented reality, remote nursing, nursing education, artificial intelligence

## Abstract

Although the metaverse is still in its early exploratory stage in the field of nursing, it is gradually demonstrating its potential in digital health and telemedicine care, transforming the traditional nurse–patient interaction model through digital tools and the internet. The “metaverse” represents the merging of two worlds into an immersive, online, virtual, connected environment in which participants actively engage with 3D content and interact using digital avatars. The metaverse entails many possibilities and challenges in attempts to introduce new methods of nursing. This technology has many applications, particularly with respect to assisting in surgery, enhancing chronic disease management, reshaping nursing education, promoting telemedicine, and facilitating psychological interventions. While obstacles may be encountered in various areas, such as trust and security, technology, legislation and regulation, the use of non-fungible tokens as a secure asset for patient data is a potential solution to these issues.

## The rise of the metaverse

The term “metaverse” was first used in Neal Stephenson’s 1992 science fiction novel Snow Crash to describe technology that uses the internet to combine the real and virtual worlds, in which our online presence is as important as our physical presence (1). In December 2021, Mark Zuckerberg, who invested considerable resources in the development of a three-dimensional virtual world network focused on social media connectivity, announced that Facebook would officially be renamed Meta to highlight its ambitious goals (2). Since that time, the concept of the metaverse has attracted considerable attention.

### What is the metaverse?

The metaverse is a digital three-dimensional (3D) environment, generated by a combination of augmented reality (AR), virtual reality (VR), and artificial intelligence (AI), in which individuals can use personalized digital avatars that mimic real-life contexts to engage in various financial and social interactions, among other types of engagement (3). This vast network of immersive virtual environments, powered by advanced technologies, leading to a significant paradigm shift in various aspects of human life, including healthcare and nursing.

The metaverse is a pioneering technology in the field of nursing practice (4, 5). The integration of VR and AR into nursing offers a simplified model for the practical application of patient care. Real-time guidance using 3D models can assist with puncture procedures and support surgical operations, improving the accuracy of punctures and enhancing surgical quality (6). Immersive training helps providers develop clinical skills (7) and emergency response capabilities. Wearable devices and lifelogging technologies enable remote health monitoring (8), thereby enhancing the continuity and personalization of care for chronic conditions and elderly individuals. Virtual hospitals and digital interactive platforms extend the spatial boundaries of nursing services, thus improving accessibility and efficiency in care delivery (9).

**FIGURE 1:**
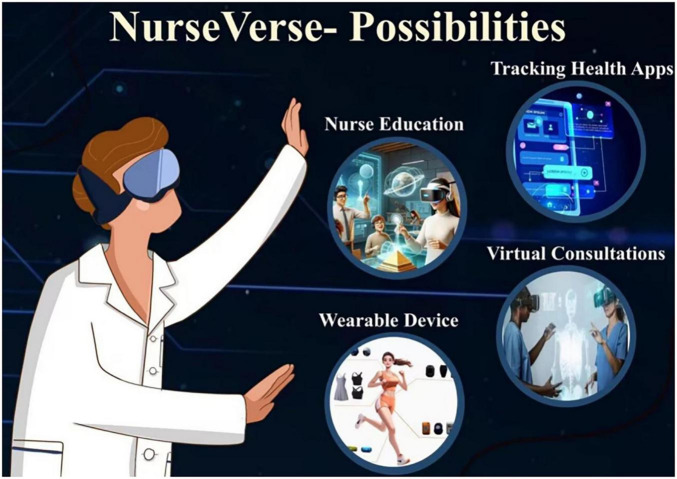
The modified NurseVerse model based on the axes and types of metaverse. The 4 types and 2 axes proposed for the metaverse can be accordingly applied to the NurseVerse concept.

Researchers have divided the metaverse into two axes (10) comprising four sections, namely, augmented reality, life log, the mirrored world, and the virtual world (Figure 1); these sections all have the potential to revolutionize digital life. In the quadrant-based introductory map to the metaverse, the vertical axis includes augmentation and simulation techniques that determine the relationship between reality and innovation. Augmentation enhances people’s sensory experiences through auditory, visual, and other sensory stimuli, thereby integrating new functions into real-world environments. In contrast, simulation of reality takes the form of a virtual world that replicates reality (1, 10). The horizontal axis includes metaverse technologies that facilitate the empirical relationship between the technology and its users. The left side reflects the external environment, whereas the right side reflects the user’s personalized experience (1, 10). The metaverse is situated within the external environment, and this external perspective is user-centered and focused on guiding the user’s interactions with these environmental features. The intimate element provides an internal perspective that focuses on technologies that guide personalized behavior on the basis of customized data.

Augmented reality (AR) is a technology that enhances the real world by overlaying virtual imaging onto it. AR is typically experienced through smart glasses, smartphones, tablets, or AR head-mounted displays (11). This technology can create realistic visualizations, such as those of vascular anatomy and subcutaneous structures (6), thereby facilitating venipuncture as well as serving as a valuable tool for emergency preparedness and training scenarios that are difficult to replicate. It supports novice doctors and nurses in performing clinically precise procedures. In mass casualty incidents, smart glasses equipped with AR have been used for triage, enabling digital documentation of triage results. Compared with traditional methods, AR approaches not only improve tactical response at the scene but also support decision-making and accelerate the overall workflow (12, 13). Furthermore, AR technology can assist in wound care management using smart glasses (14) and enhance students’ learning experiences by improving their knowledge, understanding, and practical skills—thus transforming medical and nursing education (15, 16).

Lifelogging refers to the use of smart devices—such as smartwatches, smartphones, and wearable sensors—to record one’s daily life activities via the internet or associated platforms (e.g., Instagram, health monitors) (1, 17). By combining such devices and mobile applications, users can document health-related information, including daily activities, dietary habits, step count, blood pressure, and body weight, which can help improve lifestyle behaviors (8). Wearable sensors and devices offer a more comprehensive way to track individuals’ activities and physical conditions in daily life (18). Additionally, these devices can provide essential health indicators by continuously or intermittently monitoring physiological signals such as electrocardiograms (ECGs) and electroencephalograms (EEGs). This technology is particularly valuable for issuing early warnings before health abnormalities occur, thereby supporting early diagnosis and timely treatment (19). To manage patients with chronic conditions, healthcare providers can use lifelogging data to dynamically adjust rehabilitation plans or medication regimens. Lifelogging also enhances patients’ engagement in their own health management, promoting self-care and health empowerment.

Mirror worlds represent simulations of map-based external environments (e.g., educational spaces, Google Maps, Google Earth). In the healthcare context, mirror worlds use digital technologies—such as the Internet of Things (IoT), AI, and 3D modeling—to map physical medical and nursing environments, patient data, and equipment status into virtual spaces in real time. This technique enables the construction of interactive, visualized, and analyzable digital twin systems. In China, the city of Shanghai has already established metaverse-based smart hospitals and specialty hospitals whose physical and digital hospital spaces are deeply integrated. Through digital twin technologies, physical environments are reconstructed that empower employees to achieve more efficient and intelligent nursing and medical management (9). Digital twins of human organs can facilitate personalized treatment strategies and predictive modeling of disease progression (20). However, current technologies still face challenges in accurately modeling and simulating precise biological digital twins (20).

Virtual worlds are computer-generated, persistent 3D digital environments that, when accessed through head-mounted displays, create a fully immersive online 3D reality (21). Within these environments, individuals interact, communicate, learn, entertain, and even work solely through virtual avatars; examples include online multiplayer games, virtual hospitals, and consultation rooms. In the virtual world, virtual clinics can be designed to meet both patient needs and the professional requirements of doctors and nurses. For example, when a patient is receiving consultation for liver surgery, the virtual clinic can include educational materials related to liver disease. To enhance the patient experience during waiting periods, a virtual waiting area can provide an overview of the condition, using 3D models to illustrate relevant anatomical structures, thereby helping patients better understand their health status (22). Moreover, virtual reality within virtual worlds can be used to deliver psychological interventions, thereby helping reduce patient anxiety and depression (23, 24).

The metaverse has seen widespread development across various fields. In industry, beyond employee training, the metaverse is driving significant societal advancements by enabling immersive human–machine interactions, potentially revolutionizing production and remote collaboration. The industrial metaverse seamlessly integrates within digital ecosystems, fostering engagement and innovation, and is paving the way for Industry 5.0 (25). In business, the metaverse provides shoppers with immersive shopping experiences and virtual product demonstrations within digital worlds, offering new opportunities for interactive marketing campaigns. Businesses can attract customers through virtual storefronts, showcase products in 3D, and leverage real-time data analytics to streamline supply chain operations (26). In architecture, the metaverse has numerous long-term impacts on the construction industry, such as enhancing building energy efficiency, optimizing materials and workforce management, reducing construction waste, minimizing environmental footprints, improving collaboration and communication among industry stakeholders, and significantly contributing to the circular economy through circular architecture (27). In entertainment, the metaverse enhances entertainment experiences by enabling virtual events, immersive gaming, virtual concerts, and interactive storytelling. Users can immerse themselves fully in virtual worlds, where they can engage in entertainment and social interactions with others (26). In healthcare, integrating the metaverse represents a major technological advancement with the potential to transform various aspects of medical practice. Technologies such as VR, AR, and other immersive tools can bridge the gap in clinical consultations by overcoming distance barriers, allowing patients to better understand their conditions within 3D VR environments, ultimately optimizing treatment plans and improving patient care (28).

### Developing the NurseVerse

The term NurseVerse is a fusion of “Nursing” and “Metaverse,” leveraging technologies such as VR, AR, AI, blockchain, and IoT to create an immersive and interconnected digital nursing environment. The NurseVerse specifically enables nurses to harness the power of the metaverse, allowing nursing professionals, patients, and other healthcare practitioners to engage in training, diagnosis, collaboration, and research within a virtual space. Additionally, it supports data sharing and personalized nursing services, thus introducing a groundbreaking digital world full of possibilities for the field of nursing.

**FIGURE 2:**
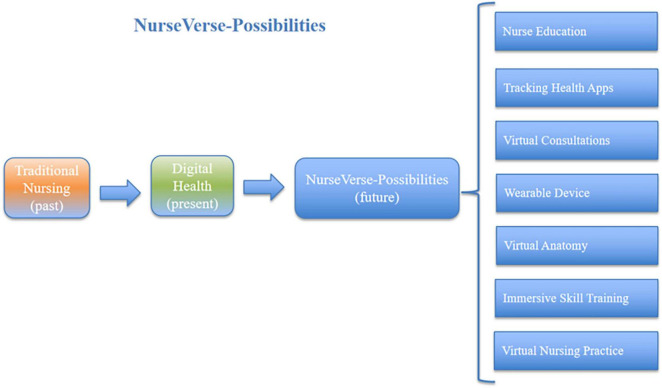
The possibilities of the NurseVerse.

**FIGURE 3:**
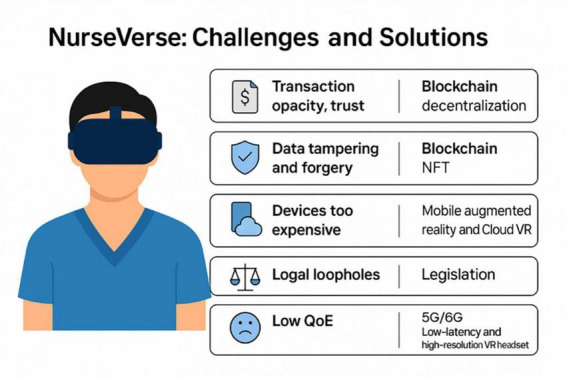
The challenges and solutions of the Nurseverse. Illustration was created using artificial intelligence. AI name: ChatGPT, model version: GPT-4.5 (GPT-4-turbo), source: Developed by OpenAI.

Although healthcare and life sciences have been slow to embrace the ability of technological change to shape the ways in which people work (29, 30), the nursing community continually explores the best ways to apply modern and innovative technologies in clinical settings (31, 32). The NurseVerse continues to evolve on the basis of existing digital health foundations, with key distinctions between the NurseVerse and current digital health solutions (Table 1). We explore the potential applications of the NurseVerse and the corresponding challenges, particularly with respect to the various benefits of this digital trend for nursing. The revised model of the nursing metaverse, which is based on 2 axes and 4 types of metaspaces, is shown in Figure 1. Initially, the possibilities and cutting-edge nursing applications offered by the nursing metaverse seem to be very broad (Figure 2); however, this technology simultaneously entails many safety, technical, and ethical issues (Figure 3).

**TABLE 1:** Differences between NurseVerse and existing digital health solutions.

Comparison dimension	NurseVerse solution	Existing digital health solutions	References
Technological foundation	Based on metaverse technologies (VR/AR, AI, blockchain)	Mainly mobile applications, remote monitoring devices, etc.	(33)
Interactivity	Immersive 3D interaction (VR/AR)	Primarily 2D interfaces (apps/videos)	(34)
Nursing education	Virtual wards, simulated patients, interactive operations	Online courses, video-based learning	(35)
Remote nursing	Nurses can interact with patients in the virtual world	Remote monitoring, video consultations	(36)
Personalization	AI-driven personalized nursing plan generation and optimization	Lower level of personalization, mostly standardized services	(34)
Operability	Nurses can simulate nursing operations in the virtual environment	Mainly data monitoring and information transmission	(37)

### Surgical assistance

In addition to existing applications of surgical robotics integrated with AR, VR and AI, the metaverse has the potential to enhance healthcare professionals’ technical skills and their awareness of potential risks and complications (34). Employing this technology—from initial patient consultation and preoperative planning to intraoperative 3D navigation and postoperative follow-up—provides patients with a comprehensive understanding of the entire surgical process (38). Simulated training in a virtual environment using VR technology is not only highly feasible in real-world contexts but also represents a vital component of future intelligent surgical collaboration systems. In virtual operating rooms, nursing students can engage in the complete workflow of a scrub nurse, including preparing sterile fields, performing preoperative “time-out” verifications, counting instruments and sharps, passing instruments, and handling surgical specimens (37). Virtual simulation provides nurses with a safe, standardized, repeatable, and highly controlled learning environment in which they can familiarize themselves with complex procedures and clinical scenarios (39), thereby significantly reducing the risk of errors during real surgeries. Moreover, virtual reality simulations offer an immersive experience that allows nursing students to interact with members of the virtual surgical team, thus enhancing intraoperative communication and teamwork skills (37).

Through medical-enhanced VR/AR (MER), vital signs, laboratory data, and imaging results can be overlaid in real time onto the visual field of healthcare professionals, enabling more accurate intraoperative decision-making. This integrated information display reduces the need for clinicians to shift their gaze between the patient and external monitoring devices, thereby enhancing situational awareness and decision-making efficiency during surgery (40). Intraoperatively, AR can project anatomical structures—such as blood vessels or organs—directly onto the patient’s body, helping surgeons locate the surgical site precisely while allowing nurses to quickly identify relevant areas of focus. This spatial visualization capability aids in accurate targeting and reduces the risk of damaging critical structures, thereby lowering the incidence of surgical errors. Currently, leading healthcare institutions have begun exploring the integration of the metaverse into clinical nursing practice. The following cases illustrate typical applications of the metaverse in real-world healthcare settings: physicians at Stanford Medical Center have integrated AR tools into surgical practice by using the Apple Vision Pro headset to visualize data in real time during procedures, which has the potential to significantly improve teamwork and collaboration within the surgical team (41).

However, virtual reality simulations (VRS) are still in the early stages of development (42), and existing research on virtual simulation presents known methodological limitations, such as small sample sizes, a lack of reliability reporting, and the limited use of validated surveys (43). Moreover, current AR systems have not yet been widely deployed in the nursing field; nevertheless, they have demonstrated clear potential in enhancing intraoperative information synchronization, optimizing task collaboration, and strengthening nurse engagement.

## Strengthening chronic disease management

The metaverse can facilitate timely and long-term follow-up for chronic diseases, and patients and nurses can meet in 3D virtual spaces using AI to provide better user experiences in the context of telemedicine care services. Regardless of the physical location of the patient (44), the nursing specialist or caregiver can conduct virtual consultations and follow up with the patient to assess the progression of the disease and understand its prognosis and outcomes. AI devices can be used to monitor the patient’s body temperature, heart rate, blood pressure, blood sugar level, and even 12-lead ECG devices (45). AI technology has been used to analyze the data collected by smart socks, thus facilitating efficient gait analysis (46). This technology can offer personalized activity guidance to patients with diabetes and obesity and suggest appropriate activity intensities and times for each individual to improve the efficiency of fat reduction efforts. Patients can use AI-based systems and wearable technology to monitor their health status 24 h a day, enabling AI to identify threats in a timely manner. Through avatars, existing remote consultations using video phones can be transformed into more engaging interactions between doctors and patients or between nurses and patients. Patients can ask doctors and nurses for relevant consultations, personalized care, and patient education without the need to make doctor appointments or visit hospitals personally; moreover, the process is highly accessible to elderly people, people living in remote areas, and people with disabilities. However, AI detection occasionally encounters anomalies, false positives, and false negatives. Therefore, healthcare professionals need to assess results in conjunction with patient symptoms, verify AI findings through multiple sources, pay special attention to vulnerable populations, and ensure data security to maximize the value of AI while minimizing its risks. In the metaverse, wearable devices provide safe and efficient data to facilitate the early detection, diagnosis and treatment of chronic diseases. The metaverse can assist in the prevention and management of chronic diseases and is highly convenient for patients with chronic diseases.

Based on the above functional descriptions and analysis of potential advantages, the following presents practical applications of the metaverse in chronic disease management. Currently, Gwangmyeong Hospital of Chung-Ang University in South Korea has utilized digital twin and metaverse technologies to create a virtual hospital called “Metaversepital.” This facility allows users to receive medical consultations and treatments without physically visiting the hospital (47), which enhances remote management capabilities for patients with chronic diseases. Constance Johnson and colleagues established the “Second Life Impacts Diabetes Education & Self-Management” (SLIDES) community on the virtual platform *Second Life*, providing education and support for individuals with type 2 diabetes (48).

## Reshaping nursing education

The adoption of the metaverse in nursing education seems to offer unlimited potential. For nurses or nursing students, because training varies by time and region, all trainees can receive the same standardized education through metatime, regardless of where or when they can access such education (49). The combined use of AR, VR and AI highlights new horizons for nursing training with respect to viewing virtual patients for the purposes of diagnosis and evaluation. The metaverse could even allow nurses to “enter” a virtual human body, thereby observing a 360-degree panoramic view of the cardiovascular system or simulating a real piercing process that could reduce the risk of accidental injury due to needles or other sharp instruments, mitigate occupational exposure and improve the safety of clinical nurses’ attempts to perform invasive procedures. Since onsite training opportunities for peripherally inserted central catheter (PICC) insertion are not always easily accessible, in light of the growing demand for such opportunities and the lack of adequate training centers, virtual education courses may be a reasonable and appropriate option in this context. In January 2022, students at Queen Mary Hospital in London observed the first lecture in the metaverse, in which context participants were connected through a VR computer app and VR headset, which offered them a more amazing and interactive experience in the metaverse than would be possible through traditional online experiences. Many students and medical practitioners reported a high level of satisfaction with this situation, which they stated had a positive effect on their virtual learning (50). The metaverse has great potential in the context of nursing training, and this hyperspace is likely to revolutionize nursing education (51).

For patients, traditional explanations using charts and flat surfaces are difficult to understand. Through the use of simple 3D animation and VR devices, patients can discover their own health problems, understand the basic information about the disease, and participate fully in treatment and care decisions, such as those regarding peripheral or central venous treatment, thereby improving patient compliance and reducing the incidence of disease. The advantages of the metaverse in the context of nursing education include safety, repeatability, universality, cost and time benefits, and the lack of any risk of harm to patients (43, 52). The implementation of nursing teaching in the metaverse is a viable pedagogical strategy that can provide collaborative opportunities to nurses, nursing students, and teachers, and its increasingly widespread use is expected to have profound impacts on nursing education.

The latest developments provide concrete examples of how the metaverse is reshaping nursing education and facilitating its translation into clinical nursing practice. The University of Florida has established simulation laboratories that integrate virtual reality technology to provide nursing students with immersive learning experiences. These labs allow students to practice scenarios such as measuring blood pressure, administering injections, changing dressings, and simulating childbirth (7). UbiSim, a company dedicated to creating immersive VR training platforms for nurses, has already implemented VR-based training for nursing professionals (53). The UbiSim platform offers a variety of virtual nursing scenarios to help students safely practice and enhance their clinical skills (54).

## Connection to telemedicine care technology

In light of the ongoing pandemic, the metaverse can allow patients to participate in consultations at home, which can enable patients in remote areas to obtain more comprehensive services of identical quality and reduce the risk of infectious diseases in medical institutions (55), which is beneficial for patients, doctors and nurses.

Current telemedicine relies primarily on video consultations in which healthcare providers and patients communicate remotely through 2D screens. While this approach overcomes geographical barriers, it offers limited interactivity and lacks a sense of realism. AI-assisted diagnostics also depend mainly on 2D medical imaging (such as X-rays, computed tomography [CT] scans, and magnetic resonance imaging [MRI]) and electronic health records for analysis. In contrast, metaverse-based healthcare enables the construction of 3D virtual consultation rooms in which healthcare providers and patients can communicate face-to-face. This immersive 3D environment enhances the realism and depth of remote medical encounters, thus allowing clinicians to assess patients’ conditions more intuitively (34). Moreover, the integration of AI with digital twin technology allows for the creation of individualized 3D physiological models of real patients. Within immersive environments, clinicians can rotate and magnify these models and interact with AI in real time, improving the precision and personalization of diagnostics (56).

In fact, following the COVID-19 pandemic, the number of healthcare facilities in the United States with the ability to provide remote services increased from 43% to 95% (57). Compared with current telemedicine care services (including telemedicine models, AI-based diagnostics, and video conferencing) or face-to-face conversations, the metaverse approach facilitates interaction in a nearly face-to-face, 3D virtual space, which enables doctors and nurses to talk with and observe patients more closely. This approach is especially useful for regular follow-up, as it allows nurses to monitor basic conditions such as blood sugar, blood pressure, and ECG parameters (21). Wearables are important in nursing because they have the potential to overcome the challenges associated with traditional nursing, in which nurses’ interactions with patients are all face-to-face and functional assessments are limited by physical barriers. The incorporation of innovations in telemedicine care into metaspaces has been recognized as a key component of a functional, concrete, and evolving NurseVerse.

Based on the above technological advantages, a growing number of applications connecting the metaverse with remote healthcare and nursing services have gradually emerged worldwide. Currently, the Shanghai Metaverse Specialty Hospital has developed metaverse + 5G glasses-free 3D slit-lamp microscopy technology for remote diagnosis and treatment (9). Chung-Ang University Gwangmyeong Hospital in South Korea has also utilized digital twin and metaverse technologies to create a virtual hospital named “Metaversepital,” enabling users to receive remote medical consultations, treatments, and nursing care (47).

## Psychological intervention therapy

Finally, the NurseVerse has unlimited potential with respect to psychological intervention therapy. Through the use of VR technology, the metaverse can greatly reduce the various psychological (such as anxiety and depression) and pain problems experienced by patients and reduce the length of their hospital stay; it can also help caregivers reduce stress and enhance their mood. The use of VR can help reduce anxiety and depression among breast and lung cancer patients (23, 24). VR-based mindfulness training can significantly reduce anxiety, depression and cancer-related fatigue among ovarian cancer patients in the context of chemotherapy (58). In a study that presented immersive VR environments such as cliffs or dream castles to 67 patients after heart surgery and used a Likert scale to evaluate the results, 90% of patients reported reduced pain levels after receiving VR treatment (59). The use of VR by burn patients has also been shown to play a positive role in relieving pain (60). VR is a safe and effective technology that offers the advantages of immersion, interactivity and conceptualization. During the COVID-19 pandemic, Min He et al. used fifth-generation plus VR (5G + VR) equipment to establish a visiting channel, and after treatment, patients’ anxiety and depression decreased significantly (61). Home care and employee stress care based on VR technology have also been demonstrated as highly desirable in normal contexts (62–64). Among intensive care unit (ICU) nurses who used VR technology to relax during breaks, 62% reported that such technology helped reduce stress (65). For patients, metaverse technology can alleviate the pain and anxiety caused by their disease. For doctors and caregivers, metaverse technology can reduce the stress of both work and life. The emergence and progress of the metaverse are highly valuable to the medical community. Despite the great potential of VR/AR technology in this field, its implementation in real-world practice requires careful consideration of its side effects. One such issue is that prolonged VR use may cause dizziness, nausea, eye fatigue, and spatial disorientation, similar to motion sickness, especially in highly immersive virtual environments (66). Additionally, most studies on VR in psychological interventions have small sample sizes and short follow-up periods, limiting the evidence of its long-term efficacy (43).

These theoretical advantages have begun to show initial results in some practical scenarios. The following applications further illustrate the use of the metaverse in alleviating psychological stress for patients and healthcare providers: Stanford University’s Lucile Packard Children’s Hospital has implemented VR technology in its pediatric medical procedures, such as intravenous insertions and wound dressing changes. By engaging children in games or exploratory activities within a virtual environment, the technology helps reduce their pain perception and anxiety levels (67). During the COVID-19 pandemic, the University Medical Center Groningen (UMCG) in the Netherlands introduced a VR application called “VRelax” for ICU nurses. This app offers various natural scenes—such as swimming with dolphins or walking through forests—and has been effective in reducing stress among nursing staff (68).

### Challenges

While the NurseVerse has the potential to advance the field of nursing, the various challenges associated with its use must also be considered. These challenges pertain to different areas, such as trust and security, technology, legislation and regulation.

### Trust and security

Although the NurseVerse shows great potential in the field of medical and nursing care, some patients remain skeptical about its application. Health data is highly sensitive information, and patients question the reliability and authenticity of the NurseVerse. They worry that doctors and nurses may be unable to assess their conditions accurately through a virtual platform, raising concerns about trust in the system. These concerns are especially pronounced among elderly patients, who often have limited adaptability to emerging technologies and may be reluctant or unable to use NurseVerse-based healthcare services.

The NurseVerse requires the storage and sharing of a large amount of patient health information. In this environment, users interact through virtual identities, which offer a more flexible and immersive experience but also introduce risks of privacy breaches and illegal activities (1). Major data breaches involving insurance companies such as Anthem (69) and Humana (70) have heightened these concerns. Hackers or malicious users may exploit virtual identities to commit fraud, engage in identity theft, or disseminate false healthcare information, thereby compromising patient safety. Moreover, the open nature of virtual healthcare platforms makes patients more vulnerable to misleading medical advice, which could result in inappropriate treatment or delays in care.

Harassment and potential abuse have already been observed in traditional social media metaverse platforms (71). Therefore, ensuring legal protection and avenues for the prosecution of such behavior in the NurseVerse will be critical. Identity theft and verification also represent key challenges in the NurseVerse context. Doctors, nurses, or their avatars must be reliably identified as legitimate and licensed healthcare professionals. This challenge highlights the need not only for mutual recognition among real humans in virtual spaces but also for the ability to distinguish between human users and avatars generated entirely by AI (72).

Blockchain (73) and non-fungible tokens (NFTs) (74) are regarded as important tools to increase patient trust, ensure the security of healthcare data, and support digital identity verification (75). Blockchain (73) is a decentralized and distributed database used to record information that can ensure data security, transparency, and immutability. It relies on cryptographic algorithms and consensus mechanisms to guarantee the authenticity and reliability of data without the need for a central authority such as a hospital or a healthcare institution’s centralized database. Traditional medical data is typically stored on servers managed by hospitals or healthcare institutions, which may lead to data silos, difficulties in data sharing between different entities, and issues of information opacity, where users cannot verify the authenticity of the data. Blockchain enables decentralized storage of patient health records, thus enabling patients to authorize different medical institutions to access their medical histories without relying on a single hospital’s database. The transparency and traceability of blockchain ensure that every access or modification of data is recorded, which can prevent tampering. Running the NurseVerse on a blockchain-based decentralized system introduces different encrypted methods for handling patient data and ensures strict compliance with medical and nursing standards in practice and processes. The decentralization, immutability, and reliability of blockchain can significantly increase patient trust and safety.

An NFT is a unique set of computer code registered on a blockchain that records the ownership of digital assets (76). NFTs can store large amounts of data, contain transaction IDs, and maintain detailed historical records, enabling them to function as certificates of authenticity or digital fingerprints for data (77). NFTs can help promote a more democratic, transparent, and efficient health information exchange (HIE) system in which patients are empowered to participate in decisions about how and with whom their personal (sometimes known as “protected”) health information (PHI) is shared (76). However, despite the enhanced data security offered by NFT and blockchain technologies, significant challenges remain. The legal and regulatory frameworks surrounding blockchain are still evolving, and unified global standards remain lacking. Additionally, blockchain consumes substantial storage and computational resources, which may lead to performance bottlenecks in healthcare scenarios that require frequent access to medical data.

### Technology

The development of the NurseVerse requires state-of-the-art technology, but the corresponding hardware is not light, portable, or inexpensive. A considerable amount of hardware, such as VR headsets, wireless heart rate and blood pressure monitors, gait analyzers, and the most up-to-date computer processors, is needed to maximize the development of the metaverse, which requires sophisticated technology that entails high costs. Economic and technological inequalities lead to health inequalities for economically disadvantaged groups and countries, and accessing metaverse services is more difficult in this context.

For users who cannot afford high-end equipment, mobile augmented reality (mobile AR) (78) and cloud-based virtual reality (cloud VR) (79) can serve as alternatives to promote digital equity. Mobile AR is a solution based on smartphones or tablets, eliminating the need for users to purchase expensive AR headsets and enabling users to access the technology anytime and anywhere. With advantages such as low cost, high portability, and ease of use, mobile AR is widely applicable in fields such as education, healthcare, and nursing (78, 80). Cloud VR uses cloud servers for rendering and computing, allowing users to access high-quality VR content through standard devices such as smartphones, tablets, or regular computers, thereby reducing the cost of use. Moreover, cloud VR offers low latency and visual continuity (81), enabling more users—especially those with limited resources, such as older adults and people in remote areas—to enjoy immersive healthcare experiences (79).

The compounded risk of cybersickness and motion sickness caused by VR devices has become a barrier to the quality of experience (QoE) for users in the metaverse (66). This issue is particularly evident in immersive settings such as virtual remote consultations, where prolonged use of head-mounted VR displays increases the likelihood of discomfort due to mismatches between visual and vestibular sensory input. Symptoms may include dizziness, nausea, reduced attention span, pain, and blurred vision (66, 82). Users can alleviate these symptoms by prioritizing the use of low-latency, high-resolution VR headsets combined with precise head- and eye-tracking technologies. These features help reduce sensory lag and screen tearing, thereby mitigating the discomfort associated with cybersickness and motion sickness. In addition, the high bandwidth and low latency offered by 5G/6G networks further enhance the immersive and seamless nature of the VR experience, ultimately improving users’ overall QoE (66).

Another challenge in the metaverse is identity theft, which can be exploited to fraudulently obtain health insurance benefits. Malicious actors may hack into personal accounts and impersonate legitimate users—such as by stealing their avatar information (83) or mimicking their behavioral traits—to fabricate identities. They might also collect identity-related data such as avatar appearances and gestures (84), using this information to create fake doctors or patients. These fake doctors can then provide false medical consultations or issue prescriptions, while impersonated patients “receive” costly healthcare services in the virtual environment—services that were never actually delivered—ultimately defrauding insurance companies.

Although various preventive measures have been introduced, such as regularly changing avatar appearances (85), splitting personal information across internet networks (83), and using biometric and digital information for authentication (86), these strategies must continually evolve alongside technological advancements. Detection technologies, including algorithms for analyzing the authenticity of potentially AI-generated content, are also emerging (87). However, such technologies not only require ongoing refinement but may also be exploited by criminals to facilitate other illicit activities (88). Given that victims and perpetrators of the same case may be located in different parts of the world (89), international cooperation is becoming increasingly vital. It is essential to address identity-related crimes in the metaverse to promote information sharing and establish unified technical standards.

### Regulation and legislation

Patients who visit the hospital can store their entire medical history, medication history, medical conditions, and allergies in their own personalized NFTs, which can only be viewed by the patient and their doctor or anyone else to whom the patient offers access. This approach can save time, effort and money for patients as well as time and human resources for hospitals. Patient conditions and data are among the most valuable assets in healthcare, and their safe use, management and sharing are continual requirements for healthcare professionals. Despite the many benefits of NFTs to both patients and hospitals, regulation and standardization remain necessary to build trust in technological enablers and test their performance in real-world settings.

Who is responsible when a patient receives inappropriate medical care? Liability is a key issue in the use of technology in healthcare (90). Metaverse providers hold responsibility for compliance with device specifications. However, when the NurseVerse becomes an integral part of the doctor–patient or nurse–patient relationship, its providers become participants in the care process. When an error occurs and a decision is made on the basis of perceptions or information obtained from the virtual world, it is essential to determine who should be held accountable. As in any other area of healthcare, doctors and nurses ultimately remain responsible for the care they provide.

Cybersecurity and patient safety must be strictly protected under the law. Although existing data privacy regulations (91) require organizations or institutions to use encryption or pseudonymization for emails, messages, notes, and cloud storage whenever feasible, to reduce the risk of patient data being misused or illegally accessed, the importance of addressing issues such as identity theft, misidentification, medical errors, and data breaches in the metaverse goes far beyond the need for technical upgrades. It is essential to establish comprehensive policies and regulatory frameworks with clear legal provisions to protect patients from harm caused by incorrect medical practices or technological vulnerabilities. Insurance companies need to revise their policies to adapt to emerging forms of healthcare in the metaverse. Medical device companies must ensure that their equipment complies with safety standards and undergoes regular security assessments. Governments must take the lead in the legislative process by coordinating input from the healthcare industry, technology companies, legal experts, and ethicists. They should formulate specialized laws for metaverse-based medical systems and establish dedicated regulatory bodies to continuously monitor and assess technologies, data usage, and medical practices. Involving all stakeholders and addressing legal and regulatory challenges will be essential to ensure safe, ethical, and effective healthcare in the metaverse.

### Limitations

Although this article systematically explores the application of metaverse in nursing practice from multiple dimensions—including surgical assistance, strengthening chronic disease management, reshaping nursing education, connection to telemedicine care technology, psychological intervention therapy—several limitations remain: as research on the metaverse in the nursing field is still in its early exploratory stage, existing studies on its practical application are limited, mostly involving small-scale implementations (9, 48, 67, 68). There is a lack of large-scale, systematic empirical data. Therefore, some of the viewpoints presented in this review need to be validated by future studies with larger sample sizes and stronger empirical designs. Nursing is a discipline that emphasizes humanistic care, emotional communication, and ethical decision-making. Certain nursing practices—such as end-of-life and palliative care, psychological care for patients with depression, support for individuals with cognitive impairments and communication during acute clinical deterioration—are difficult to replicate or replace fully through virtual means. While this review discusses the technical advantages of the metaverse, it does not delve deeply into its impact on the humanistic and ethical aspects of nursing care, nor into how to strike a balance between technology and compassion. There are significant differences in the acceptance of metaverse technologies among nurses and patients of different ages, cultural backgrounds, and levels of digital literacy. This review does not offer a detailed analysis of how such differences may affect the implementation and scalability of the metaverse in nursing settings. Furthermore, the readiness of the nursing profession to adopt metaverse-based tools varies considerably. While some departments and professionals demonstrate enthusiasm, others remain cautious due to concerns about the maturity of the technology and associated practical challenges. Although components of the metaverse—such as virtual reality, augmented reality, artificial intelligence, and digital twins—have shown early development at the technical level, their large-scale application in real-world nursing practice still faces barriers including cost, hardware requirements, data privacy, and ethical considerations. This review does not comprehensively assess how these real-world constraints might limit the effectiveness of implementation. The full realization of a metaverse-supported nursing ecosystem remains a goal for the future.

## Conclusion

In summary, owing to the development of the core technologies associated with metaverse-augmented reality, life logs, mirrored worlds, and virtual worlds, the entire world is undergoing a digital healthcare revolution that can introduce an extraordinary dimension to the nursing environment for both nurses and nursing students. Although the application of the metaverse in nursing is still in its early exploratory stage, it has the potential to effectively complement traditional nursing approaches and may offer patients more options. A variety of AI models often serve as the foundation and fundamental components of the metaverse innovation model, and the NurseVerse has great potential to change the model of care by utilizing cutting-edge AI technologies; however, the transition from AI to the NurseVerse is not immediate, and further research on the ethical and credibility issues that emerge in this context is needed. Only by finding solutions to the issues associated with the process of adapting care to the NurseVerse in an evidence-based and ethically responsible way can we truly generate positive impacts on both individual and public health.
